# 

**Published:** 1887-05

**Authors:** 


VOLUME 3 of this JOURNAL will begin with JULY NUMBER. It starts out under very flattering auspices, with an increased and rapidly increasing circulation. It will be enlarged, and will contain one-third more of reading matter. *Begin your subscription with the new Volvme.* Portrait illustrations of prominent Texas Physicians will be a new and pleasing feature.

A RARE OPPORTUNITY FOR ADVERTISERS. Low rates—send for them.

F. E. DANIEL,
Ed. and Publisher.

